# Antibody Responses After BA.5/BF.7 Breakthrough Infection in People Living with HIV

**DOI:** 10.3390/vaccines14040339

**Published:** 2026-04-11

**Authors:** Ying Liu, Zhaowei Guo, Zhuo Yang, Yaruo Qiu, Xinglin Li, Xin Li, Leidan Zhang, Danying Chen, Xuesen Zhao, Hongxin Zhao

**Affiliations:** 1 Clinical Center for HIV/AIDS, Beijing Ditan Hospital, Beijing 100015, China; liuying9509@163.com (Y.L.); leaxin@ccmu.edu.cn (X.L.); zhangleidanzld@163.com (L.Z.); 2 Beijing Key Laboratory of Emerging Infectious Diseases, Institute of Infectious Diseases, Beijing Ditan Hospital, Capital Medical University, Beijing 100015, China; guozhaowei2024@163.com (Z.G.); zhuoyang1123@163.com (Z.Y.); lixl0514@163.com (X.L.); chendanying@ccmu.edu.cn (D.C.); 3 Beijing Key Laboratory of Tumor Systems Biology, Peking University Health Science Center, Beijing 100191, China; 2011210686@bjmu.edu.cn

**Keywords:** HIV, SARS-CoV-2, antiretroviral therapy, neutralizing antibody, antibody-dependent cellular cytotoxicity, breakthrough infection, immune imprinting

## Abstract

**Background:** People living with HIV (PLWH) constitute a vulnerable population during the COVID-19 pandemic; however, it remains uncertain whether long-term suppressive antiretroviral therapy (ART) restores sufficient immune competence to support robust hybrid immunity. While vaccination followed by breakthrough infection—termed hybrid immunity—typically elicits potent humoral responses in immunocompetent individuals, the functional quality and breadth of these responses against evolving Omicron subvariants remain poorly characterized in PLWH. This study aimed to assess functional antibody responses, including neutralizing activity and Fc effector functions, in vaccinated and unvaccinated PLWH who experienced breakthrough infection with Omicron subvariants BA.4/5 or BF.7. **Methods:** We enrolled three cohorts between December 5 and December 20, 2022: 25 HIV-negative individuals with breakthrough infection (BTI-HC), 20 ART-experienced PLWH with breakthrough infection following three-dose COVID-19 vaccination (BTI-HIV), and 10 ART-experienced PLWH with primary infection without prior vaccination (PI-HIV). All HIV-positive participants were receiving suppressive ART with regimens based on non-nucleoside reverse transcriptase inhibitors or integrase strand transfer inhibitors for a median of 3.4 years. We measured receptor-binding domain (RBD)-specific IgG, neutralizing antibody titers against ancestral D614G, Delta, BA.1, BA.4/5, BF.7, XDV, KP.2, and KP.3 variants, and antibody-dependent cellular cytotoxicity (ADCC) responses. **Results:** Despite lower absolute CD4^+^ T cell counts, BTI-HIV participants mounted RBD-binding IgG, neutralizing antibody, and ADCC responses that were comparable to BTI-HC and significantly exceeded PI-HIV across all tested variants. Both breakthrough infection cohorts exhibited immunological imprinting, with higher neutralizing titers against ancestral D614G than infecting BA.4/5 or BF.7 variants. Emerging variants XDV, KP.2, and KP.3 demonstrated substantial neutralization escape in all groups. PI-HIV showed markedly diminished neutralization breadth and failed to generate enough responses against all tested Omicron strains. **Conclusions:** Suppressive ART enables PLWH to mount hybrid immunity—conferred by vaccination followed by BF.7 or BA.4/5 breakthrough infection—with neutralizing and ADCC responses comparable to HIV-negative individuals, and significantly exceeding those of unvaccinated PLWH with primary infection. This underscores the critical role of vaccination in establishing effective hybrid immunity in this population. However, we observed immunological imprinting, with higher titers against ancestral strains than against infecting variants, and substantial escape by emerging sublineages XDV, KP.2, and KP.3 across all groups. These findings support prioritizing updated variant-containing vaccines for HIV-positive populations and reinforce the essential role of vaccination in this vulnerable group.

## 1. Introduction

The COVID-19 pandemic, caused by severe acute respiratory syndrome coronavirus 2 (SARS-CoV-2), has posed unprecedented challenges to global public health since its emergence in late 2019 [1]. People living with human immunodeficiency virus (HIV) (PLWH) represent a particularly vulnerable population in the context of the COVID-19 pandemic. HIV infection is characterized by progressive depletion of CD4^+^ T cells, immune dysregulation, and chronic immune activation, even in individuals receiving suppressive antiretroviral therapy (ART) with undetectable viral load (<50 copies/mL) for at least 6 months [2]. These immunological perturbations have been associated with impaired responses to various vaccines, including those against influenza virus, hepatitis B virus, and SARS-CoV-2 [3,4,5]. Early in the COVID-19 pandemic, PLWH were identified as being at increased risk of severe disease, hospitalization, and mortality following SARS-CoV-2 infection, particularly those with advanced immunosuppression or uncontrolled viremia [6].

The introduction of effective ART has transformed HIV from a fatal illness into a manageable chronic condition for millions of individuals worldwide. Modern ART regimens can suppress plasma HIV RNA to undetectable levels and facilitate substantial immune reconstitution, with many treated individuals achieving CD4^+^ T cell counts approaching or within the normal range. However, whether this restored immunocompetence extends to robust responsiveness to COVID-19 vaccines, and how this compares to antibody responses in HIV-negative individuals, remains incompletely characterized. Furthermore, the impact of HIV infection on the quality of functional antibody responses, including neutralizing activity and Fc-mediated effector functions, in the context of breakthrough infection with highly immune-evasive Omicron subvariants requires clarification.

Breakthrough infections in vaccinated individuals have become increasingly common following the emergence of Omicron variants [7], yet these infections may effectively act as booster immunizations, termed as hybrid immunity, potentially enhancing and broadening humoral immunity. In HIV-negative individuals, breakthrough infection has been shown to induce robust recall responses, even though there is evidence of immunological imprinting, which refers to the phenomenon where prior vaccine exposure biases the specificity of subsequent antibody responses towards ancestral antigens [8]. Whether PLWH can similarly benefit from breakthrough infection as an immunological boost, or whether HIV-associated immune dysregulation compromises this response, has important implications for vaccine policy and clinical management in this population.

Conversely, PLWH who experience primary SARS-CoV-2 infection without prior vaccination may be at particular risk of poor outcomes and impaired immune priming. Studies conducted earlier in the pandemic suggested that PLWH mounted attenuated antibody responses to primary infection with ancestral SARS-CoV-2 strains [9]. Whether this impairment persists in the era of effective ART and contemporary Omicron variants and how it compares to vaccine-boosted responses in the same population remain critically important for guiding public health recommendations.

In this study, we comprehensively characterized SARS-CoV-2-specific humoral immune responses in three distinct cohorts: HIV-negative individuals with breakthrough infection (BTI-HC), PLWH with breakthrough infection following three-dose vaccination (BTI-HIV), and PLWH with primary infection without prior vaccination (PI-HIV). All infections were caused by BA.4/5 or BF.7 variants during the same epidemic wave, enabling direct comparison of immune outcomes. We evaluated not only binding antibody titers but also neutralizing activity against a broad panel of variants spanning the antigenic evolution of SARS-CoV-2, as well as antibody-dependent cellular cytotoxicity (ADCC) as a measure of Fc-effector function. Our findings demonstrate that suppressive ART enables people living with HIV to mount robust functional antibody responses through hybrid immunity—conferred by COVID-19 vaccination followed by BF.7 or BA.4/5 breakthrough infection—that are comparable to those observed in HIV-negative BTI individuals. In contrast, primary Omicron infection in the absence of prior vaccination elicits profoundly impaired humoral immunity, underscoring the critical importance of vaccination for establishing effective hybrid immunity in this vulnerable population. Furthermore, we identify evidence of immunological imprinting in both breakthrough infection cohorts and document substantial neutralization escape by recently emerged Omicron subvariants XDV, KP.2, and KP.3. These findings inform strategies for vaccine optimization in HIV-positive populations, supporting the prioritization of updated variant-containing vaccines to enhance hybrid immunity against evolving SARS-CoV-2 variants.

## 2. Materials and Methods

### 2.1. Ethics Statement and Sample Collection

This is an observational cross-sectional study of immune response of BA.5/BF.7 BTI in PLWH. It was approved by the Research Ethics Board of Beijing Ditan Hospital (No.2022-084). All participants in the original study gave written informed consent in accordance with the Declaration of Helsinki. To understand whether HIV status might influence the immunological responses upon SARS-CoV-2 BTI, we recruited 55 COVID-19 convalescents, including 25 HIV negative participants (BTI-HC) and 20 PLWH (BTI-HIV) who received 3 doses of the inactivated SARS-CoV-2 vaccines, and 10 PLWH with SARS-CoV-2 primary infection (PI) who had received no vaccine (PI-HIV) at the Capital Medical University, Beijing Ditan Hospital, Beijing, China, between 5 December, and 20 December 2022. Although variant-specific sequencing was not performed individually, these cases were epidemiologically presumed to be BA.4/5 or BF.7 infections, as they occurred during the specific pandemic wave (December 2022–January 2023) when these sublineages were overwhelmingly dominant in the region [10]. Participants received three doses of inactivated SARS-CoV-2 vaccine: primary series (doses 1 and 2, administered at least 21 days apart) and booster dose (administered at least 6 months after dose 2). PLWH were eligible if they met the following inclusion criteria: (1) 18–60 years old; (2) have been receiving a stable ART regimen for at least 2 years with a HIV viral load ≤ 50 copies/mL; (3) had no prior SARS-CoV-2 infection before 7 December 2022; and (4) signed written informed consent. To minimize information and misclassification biases, demographic characteristics, detailed vaccination history (including exact dates and number of doses), and prior SARS-CoV-2 infection status were rigorously verified through official electronic medical records and the national digital health registry, rather than relying solely on self-report. The HIV-negative control group was matched for age and sex to the BTI-HIV group. Participants with a history of prior SARS-CoV-2 infection were excluded. Participant SARS-CoV-2 infection was confirmed by positive nucleic acid test (NAT) or rapid antigen test (RAT) documented in electronic health records, with symptom onset between 5 and 20 December 2022. Visits between days 26 and 35 after SARS-CoV-2 infection comprised the main study timepoint for immunological analysis.

### 2.2. Measurement of Omicron Receptor-Binding Domain (RBD) IgG Antibody

The anti-BA.5 RBD IgG titers in plasma were determined using an ELISA kit (kit040A, SinoBiological, Beijing, China). Briefly, for safety considerations, the plasma samples were first heat-inactivated at 56 °C for 30 min. Then, 96-well plates coated with BA.5 RBD-specific antigens were incubated with serially diluted plasma samples (8 dilutions in a 2-fold step-wise manner, commencing with a 500-fold dilution). Following incubation and washing, the plates were incubated with HRP-conjugated mouse anti-human IgG at a 1:150 dilution. Subsequently, the substrate solution and stop solution were added sequentially, and the optical density (OD) was measured at 450 nm using a microplate reader (Molecular Devices, San Jose, CA, USA). We utilized manufacturer-provided positive and negative controls. Additionally, pooled convalescent sera from COVID-19 recovered patients (positive control, OD450 > 1.0) were included as internal QC in each assay run. Pre-pandemic plasma samples were simultaneously evaluated as negative controls to establish the cut-off value (OD450 < 0.15). The endpoint antibody titer was defined as the highest plasma dilution yielding an OD value greater than the established cut-off.

### 2.3. Cell Transfection and Pseudotyped Virus Production

Pseudoviruses were generated using a vesicular stomatitis virus (VSV) backbone with the G gene replaced by luciferase (VSV-ΔG-G*-Luc, Kerafast #EB0011) and bearing SARS-CoV-2 spike proteins from the respective variants (D614G, Delta, BA.1, BA.4/5, BF.7, XDV, KP.2, or KP.3). The VSV backbone is identical across all pseudoviruses, with variant-specific differences conferred solely by the spike protein insert. Briefly, HEK-293T cells were transfected with the plasmids encoding different S proteins. After 6 h of transfection, VSV-ΔG-G*-Luc pseudovirus was added, and after 24 h of transfection, the supernatant was replaced with fresh complete Dulbecco’s modified Eagle medium. Supernatants were collected at 48 h and 72 h after transfection, passed through a 0.45 μm filter, aliquoted, and stored at −80 °C.

### 2.4. Pseudovirus-Based Neutralization Assay

The neutralization assay was performed as previously described [11]. The 50% tissue culture infectious dose (TCID_50_) of the pseudovirus was calculated using the Reed–Muench method in advance. Plasma samples should be inactivated in a water bath at 56 °C for 30 min. Serially diluted samples (5 dilutions in a 3-fold step-wise manner, commencing with a 30-fold dilution) were incubated with equivalent pseudovirus (13,000 TCID_50_/mL) in duplicate for 1.5 h at 37 °C. Thereafter, 2 × 10^4^ freshly trypsinized TREX-293 ACE2 cells were added to each well of the 96-well plate. After 18 h of incubation at 37 °C with 5% CO_2_, the luciferase substrate was added for chemiluminescence detection. The neutralization rate (%) was calculated as follows:

Neutralization rate (%) = (RLU pseudovirus − RLU pseudovirus with plasma) ×  100%/(RLU pseudovirus − RLU blank)

Neutralizing antibody titers are indicated by the plasma dilution needed to reach 50% pseudovirus neutralization (pVNT_50_). The pVNT_50_ value was derived from the neutralization curves and calculated using the log (inhibitor) versus normalized response-variable slope fit, employing the automatic outlier detection feature of GraphPad Prism software. Plasma samples with NT_50_ ≥ 30 were considered serologically positive for neutralizing antibodies.

### 2.5. ADCC Measurement

ADCC activity was evaluated with Jurkat-FcγRIIIa-NFAT-Luc reporter cells. This ADCC assay measures the ability of plasma to activate the NFAT (nuclear factor of activated T cells) pathway through FcγRIIIa in the presence of target antigens expressed on the 293T cell surface. To obtain SARS-CoV-2 S protein-overexpressing cells, 293T cells were transfected with plasmids encoding the S protein with N-terminal Myc tagged from the D614G, Delta, BA.4/5, or BF.7 variant using Lipofectamine 3000 (Invitrogen, Carlsbad, CA, USA). Target cells (2 × 10^5^ cells per well) were incubated with 1:100 dilutions of plasma samples at 37 °C for 1 h. ADCC effector cells (2 × 10^5^ cells per well) were subsequently introduced to each well. The relative light unit (RLU) was recorded after incubating for 18 h at 37 °C, as per the instructions from the GloMax^TM^ 96 Microplate Luminometer (Promega, Madison, WI, USA). To ensure assay validity, stringent quality controls were included on each plate: a characterized anti-SARS-CoV-2 positive control (pooled plasma from triple-vaccinated healthcare workers collected 2 weeks post-booster); pre-pandemic healthy human plasma as a negative control; and a baseline control containing only effector and target cells without plasma. Fold of induction was calculated as follows: RLU (induced − background)/RLU (no plasma control − background). A fold of induction greater than 2 is considered positive.

### 2.6. Statistics

Geometric mean titers (GMTs) with 95% confidence interval (95% CIs) were calculated using GraphPad Prism 9.0. Given the limited sample sizes of our predefined clinical cohorts (*n* = 25, 20, and 10), non-parametric comparative tests were utilized as the primary statistical approach. Mann–Whitney and Wilcoxon tests were used for unmatched and paired samples, respectively. Fisher’s exact test was used to analyze categorical outcomes.

## 3. Results

### 3.1. Study Design and Participant Characteristics

This study enrolled three distinct cohorts: 25 healthy controls with breakthrough SARS-CoV-2 infection (BTI-HC), 20 people living with HIV (PLWH) with breakthrough infection (BTI-HIV), and 10 PLWH with primary infection (PI-HIV). All participants presented with mild COVID-19, showing no evidence of pneumonia and requiring no specific treatment for SARS-CoV-2 infection. All HIV-positive participants had CD4^+^ T-cell counts ≥350 cells/µL at enrollment.

**Figure 1:**
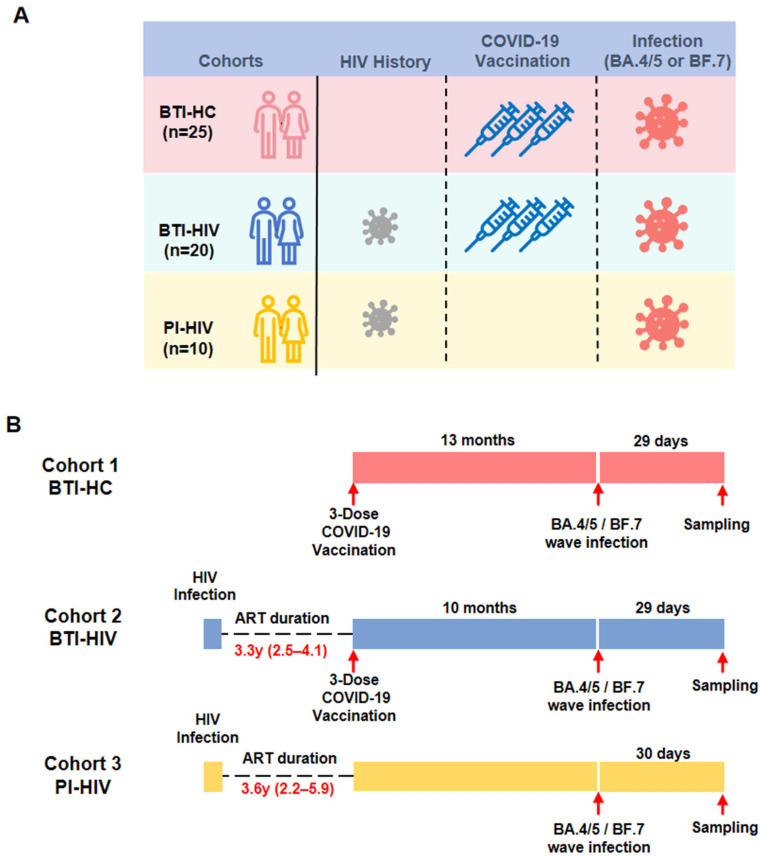
Schematic of study timeline. Plasma samples were obtained from three panels of participants. (**A**) The healthy control cohort (BTI-HC, *n* = 25, red) consisted of HIV-negative individuals who experienced breakthrough infection with BA.4/5 or BF.7 SARS-CoV-2 Omicron sublineages after receiving three doses of COVID-19 vaccine. The HIV-positive breakthrough infection cohort (BTI-HIV, *n* = 20, blue) comprised individuals living with HIV who received three-dose COVID-19 vaccination and subsequently had a breakthrough infection with BA.4/5 or BF.7. The HIV-positive primary infection cohort (PI-HIV, *n* = 10, yellow) contained individuals living with HIV who were infected with BA.4/5 or BF.7 without prior COVID-19 vaccination. (**B**) The median duration of antiretroviral therapy (ART) in the HIV-positive cohorts (BTI-HIV and PI-HIV) was 3.3 years (range: 2.5–4.1 years) and 3.6 years (range: 2.2–5.9 years), respectively. The plasma was sampled ~29–30 days after BA.4/5/BF.7 infection.

The BTI-HC cohort comprised HIV-negative individuals who experienced breakthrough infection with BA.4/5 or BF.7 sublineages following three doses of COVID-19 vaccine. The BTI-HIV cohort consisted of ART-experienced PLWH who received three-dose COVID-19 vaccination prior to breakthrough infection with BA.4/5 or BF.7. The PI-HIV cohort included PLWH who were infected with BA.4/5 or BF.7 without prior COVID-19 vaccination. Demographic and clinical characteristics were balanced across groups. Healthy controls were matched to the BTI-HIV group by age, gender, and vaccine manufacturer. The median CD4^+^ T cell counts were comparable between the BTI-HIV and PI-HIV groups [551 cells/μL (IQR: 474–636) vs. 503 cells/μL (IQR: 475–630), respectively]. All HIV-positive participants were receiving suppressive ART, with comparable median durations between the BTI-HIV and PI-HIV groups [3.3 years (IQR: 2.5–4.1) vs. 3.6 years (IQR: 2.2–5.9), respectively]. Regarding vaccination history, the median interval between the third vaccine dose and SARS-CoV-2 infection was significantly longer in the BTI-HC group compared to the BTI-HIV group [13 months (IQR: 10–13) vs. 10 months (IQR: 9–11), respectively; *p* < 0.05]. The plasma was sampled ~29–30 days after BA.4/5/BF.7 infection (Figure 1, Table 1).

### 3.2. SARS-CoV-2 RBD-Specific Antibody Responses Among Different Cohorts

**Figure 2:**
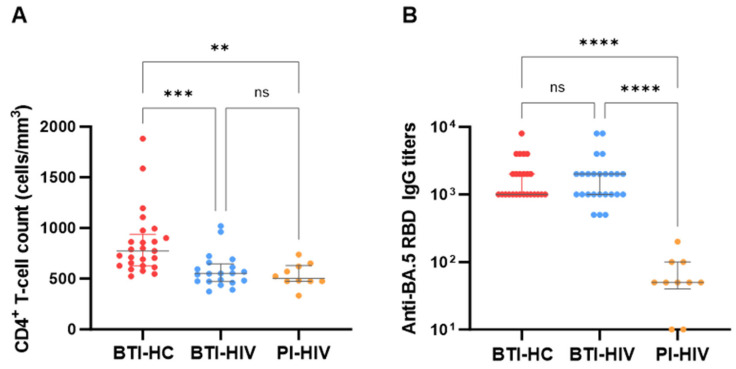
Comparison of SARS-CoV-2-specific antibody responses among different cohorts. (**A**) Scatter dot plot showing CD4^+^ T cell count (per mm^3^) at the time of sampling. Individual data points with median values (horizontal lines) and interquartile ranges for CD4^+^ T cell counts in three study groups are shown. BTI-HC (red), BTI-HIV (blue), and PI-HIV (orange). (**B**) The titer of RBD-binding IgG against BA.4/5 RBD in the three cohorts. Median titers are shown above each set of points, and the error bars indicate the 95% confidence intervals (CI). Statistical significance was determined using the Kruskal–Wallis test with Dunn’s multiple comparisons, with brackets indicating significant differences between groups (** *p* < 0.01; *** *p* < 0.001; **** *p* < 0.0001; ns, not significant).

We first assessed the immune status of HIV-positive participants at the time of SARS-CoV-2 infection. Despite prolonged antiretroviral therapy [median 3.3 years, (IQR: 2.5–4.1) for BTI-HIV and median 3.6 years, (IQR: 2.2–5.9) for PI-HIV], CD4^+^ T cell counts in both BTI-HIV and PI-HIV groups remained significantly lower than those in healthy controls [median 551 cells/μL (IQR: 474–636) and 503 cells/μL (IQR: 475–630) vs. [median 774 cells/μL (IQR: 629–939)] for BTI-HC; Figure 2A]. Notably, while all HIV-positive individuals achieved CD4^+^ T cell counts above the threshold of 350 cells/μL, indicating successful immune reconstitution with suppressive ART, their absolute CD4^+^ T cell levels still failed to reach the normal range observed in HIV-negative individuals.

We next evaluated the impact of HIV infection and vaccination status on humoral immune responses to SARS-CoV-2. Strikingly, RBD-binding IgG antibody titers against BA.4/5 were comparable between the BTI-HIV and BTI-HC groups [median: 2000 vs. 1000; *p* > 0.05], despite the significantly lower CD4^+^ T cell counts in the HIV-positive cohort (Figure 2B). This finding demonstrates that prior COVID-19 vaccination effectively primed the humoral immune system in PLWH, enabling robust antibody responses upon breakthrough infection that matched those of healthy controls. In contrast, PI-HIV participants who lacked vaccine-induced priming exhibited markedly diminished RBD-binding IgG titers (median: 50) compared to both BTI-HIV and BTI-HC groups (*p* < 0.001 and *p* < 0.01, respectively), highlighting the critical importance of vaccination in eliciting adequate antibody responses in this population.

Collectively, these results indicate that standardized ART effectively restores CD4^+^ T cell counts to levels sufficient for eliciting antibody responses in PLWH through hybrid immunity, conferred by COVID-19 vaccination followed by BF.7 or BA.4/5 breakthrough infection. However, without prior vaccination, primary SARS-CoV-2 infection in ART-treated individuals elicits substantially impaired antibody responses, underscoring the essential role of COVID-19 vaccination in protecting HIV-positive populations against circulating Omicron subvariants.

### 3.3. Neutralizing Antibody and Antibody-Dependent Cellular Cytotoxicity (ADCC) Responses Against SARS-CoV-2 Variants

To comprehensively characterize the functional antibody profiles elicited by vaccination and infection, we measured both neutralizing antibody titers and ADCC activity against a panel of SARS-CoV-2 variants, including the ancestral D614G strain, Delta, and Omicron sublineages BA.4/5 and BF.7.

**Figure 3:**
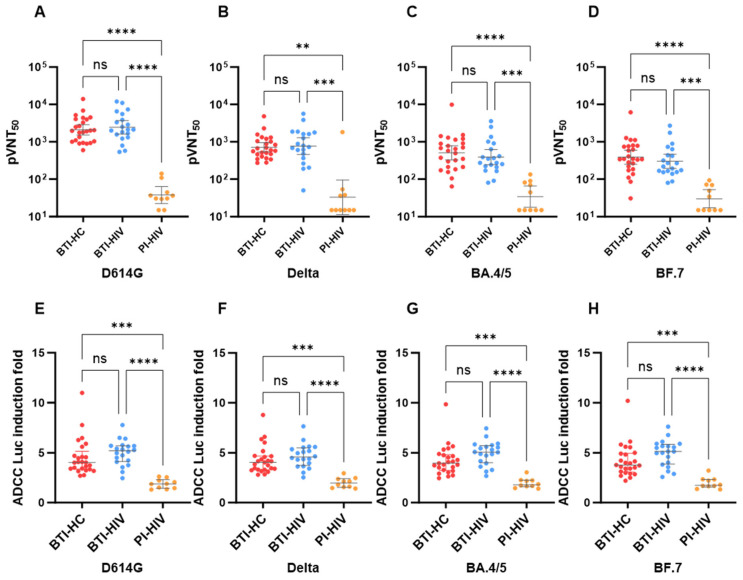
Comparison of SARS-CoV-2-specific neutralizing antibody titers and antibody-dependent cellular cytotoxicity (ADCC) responses against SARS-CoV-2 variants in three cohorts. Scatter dot plots show neutralizing antibody titers (50% neutralization titer, pVNT_50_) against variants (**A**) D614G, (**B**) Delta, (**C**) BA.4/5, and (**D**) BF.7. Geometric mean titers (GMTs) are indicated above each data set, with error bars representing 95% confidence intervals (CI). ADCC antibody responses against (**E**) D614G, (**F**) Delta, (**G**) BA.4/5, and (**H**) BF.7 are shown as fold induction mediated by FcγRIIIa-expressing cells. The median fold of induction is indicated above each data set, with error bars representing the 95% CI. Dotted horizontal lines indicate the limit of detection (LOD) (30). pVNT_50_ values below the quantitative range but still within the qualitative range (i.e., partial inhibition is observed but a dose–response curve cannot be fit because it does not sufficiently span the pVNT_50_) was counted as half (15) of the limit of LOD in statistical analysis. Statistical significance was determined using the Kruskal–Wallis test with Dunn’s multiple comparisons post hoc test. (** *p* < 0.01; *** *p* < 0.001; **** *p* < 0.0001; ns, not significant).

Consistent with the RBD-binding IgG data, prior vaccination substantially enhanced neutralizing antibody responses against all tested variants in both HIV-negative and HIV-positive individuals. BTI-HC and BTI-HIV groups exhibited robust and comparable neutralizing activity against D614G [geometric mean titers (GMT): 2098 vs. 2443; *p* > 0.05], Delta (GMT: 712.7 vs. 770.4; *p* > 0.05), BA.4/5 (GMT: 507.3 vs. 390.6; *p* > 0.05), and BF.7 (GMT: 389.2 vs. 302.9; *p* > 0.05) (Figure 3A–D). In stark contrast, PI-HIV participants mounted significantly diminished neutralizing responses across all variants compared to both breakthrough infection cohorts (all *p* < 0.001). Notably, neutralizing titers against Omicron subvariants BA.4/5 and BF.7 were markedly reduced relative to ancestral D614G and Delta strains in all groups, reflecting the extensive immune evasion properties of these contemporary variants. However, the magnitude of this Omicron-specific neutralization escape was comparable between BTI-HC and BTI-HIV groups, suggesting that vaccination preserved the qualitative breadth of neutralizing responses despite HIV-associated immune perturbations.

We further assessed the capacity of variant-specific antibodies to mediate FcγRIIIa engagement, a surrogate marker for ADCC functionality. Similar to neutralization profiles, ADCC responses against D614G, Delta, BA.4/5, and BF.7 were significantly elevated in BTI-HC and BTI-HIV groups compared to PI-HIV (Figure 3E–H). Importantly, no significant differences were observed in ADCC induction folds between the BTI-HC and BTI-HIV cohorts across all tested variants [median fold: 4.03 vs. 5.20 for D614G; 4.05 vs. 4.57 for Delta; 3.95 vs. 5.06 for BA.4/5; 3.78 vs. 5.13 for BF.7; all *p* > 0.05]. These findings demonstrate that three-dose COVID-19 vaccination effectively primed Fc-mediated effector functions in PLWH, achieving levels indistinguishable from those in healthy controls. Conversely, primary infection in ART-treated HIV-positive individuals elicited markedly compromised ADCC responses, with median induction folds substantially lower than both the BTI-HC and BTI-HIV groups (*p* < 0.01).

Collectively, these data reveal that standardized suppressive ART enables PLWH to mount neutralizing antibody responses and ADCC function through hybrid immunity—conferred by COVID-19 vaccination followed by BF.7 or BA.4/5 breakthrough infection—that are comparable to those observed in HIV-negative BTI individuals. The convergence of neutralizing and ADCC responses in the BTI-HC and BTI-HIV groups, despite lower CD4^+^ T cell counts in the latter, underscores the resilience of vaccine-primed humoral immunity in the context of well-controlled HIV infection. However, the profound functional antibody impairment observed in PI-HIV highlights the critical necessity of vaccination for protecting HIV-positive populations against severe COVID-19, particularly in the era of highly immune-evasive Omicron subvariants.

### 3.4. Neutralizing Antibody Responses Against Emerging SARS-CoV-2 Variants and Immunological Imprinting

To assess the breadth and durability of neutralizing responses against antigenically distinct SARS-CoV-2 variants, we extended our analysis to include not only ancestral D614G, Delta, and early Omicron sublineages (BA.1, BA.4/5, BF.7), but also emerging variants XDV, KP.2, and KP.3, which have recently gained epidemiological dominance.

**Figure 4:**
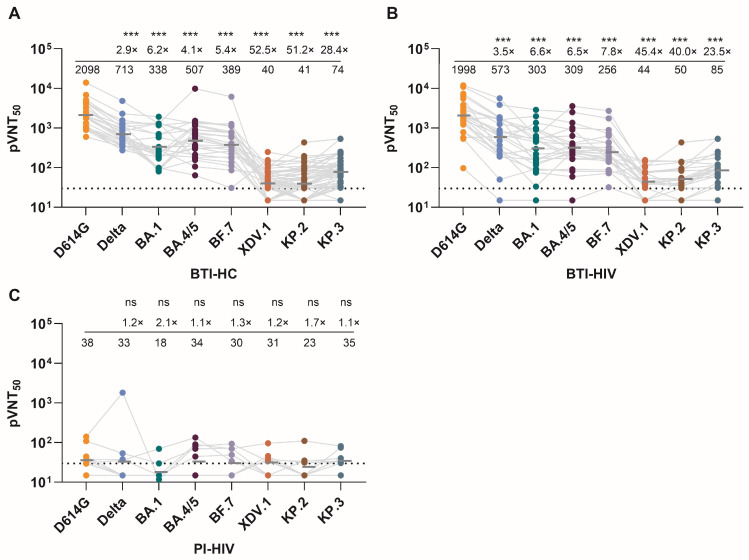
Comparison of pVNT_50_ against all tested SARS-CoV-2 variants in three cohorts used in this study. pVNT_50_ against pseudotyped viruses of SARS-CoV-2 D614G, Delta, Omicron BA.1, BA.4/5, BF.7, XDV, KP.2, and KP.3 variants were measured in three cohorts. BTI-HC (**A**), BTI-HIV (**B**), and PI-HIV (**C**). Data are presented as geometric mean titers (GMTs) with 95% confidence intervals (CI). Dotted horizontal lines indicate the limit of detection (LOD) (30). pVNT_50_ values below the quantitative range but still within the qualitative range (i.e., partial inhibition is observed but a dose–response curve cannot be fit because it does not sufficiently span the pVNT_50_) was counted as half (15) of the limit of LOD in statistical analysis. Fold reduction in neutralization potency of each variant relative to the ancestral D614G strain within each cohort. Statistical significance was determined using the Kruskal–Wallis test with Dunn’s multiple comparisons. (*** *p* < 0.001; ns, not significant).

The BTI-HC and BTI-HIV groups exhibited broad neutralizing activity against ancestral and early variants, with GMTs against D614G and Delta significantly exceeding those against Omicron sublineages (Figure 4A). Strikingly, both breakthrough infection cohorts showed substantial cross-neutralization of BA.4/5 and BF.7—the variants responsible for their infections—despite these being antigenically distant from the vaccine strain. In contrast, PI-HIV participants mounted uniformly low neutralizing responses across the entire variant panel, with GMTs consistently near or below the limit of detection for all tested viruses (Figure 4C). These findings reinforce that vaccination is indispensable for generating variant-cross-reactive neutralizing antibodies in PLWH, whereas primary infection alone, even in the context of suppressive ART, fails to elicit enough humoral immunity against diverse SARS-CoV-2 lineages.

A notable finding was the observation of immunological imprinting in both the BTI-HC and BTI-HIV groups. Despite being infected with BA.4/5 or BF.7, participants in these cohorts exhibited higher neutralizing titers against the ancestral D614G vaccine strain than against the infecting Omicron variant itself. Quantitative analysis revealed that neutralization potency against BA.4/5 and BF.7 was reduced by approximately 4.1-fold and 5.4-fold, respectively, compared to D614G in BTI-HC (Figure 4A), corresponding to an antigenic distance of 2.0 and 2.4 Antigenic Units (AU), respectively [AU = log_2_(fold-reduction)], with comparable reductions observed in BTI-HIV (Figure 4B). This pattern (termed “antigenic sin” or immunological imprinting) suggests that the immune response in vaccinated individuals remains biased toward the original vaccine antigen, even after breakthrough infection with antigenically divergent variants [8,12]. Importantly, the magnitude of this imprinting effect was indistinguishable between the BTI-HC and BTI-HIV groups, indicating that HIV infection does not exacerbate vaccine-induced imprinting in individuals receiving effective ART.

We next evaluated neutralizing activity against contemporary variants XDV, KP.2, and KP.3. All three groups showed markedly diminished neutralization against these latest variants, with GMTs approaching or falling below the limit of detection (Figure 4A). BTI-HC and BTI-HIV maintained marginally higher titers against XDV, KP.2, and KP.3 compared to PI-HIV, but the absolute neutralizing activity was substantially compromised across all cohorts. Fold-reduction analysis demonstrated that XDV, KP.2, and KP.3 evaded neutralization by 52.5-fold, 51.2-fold, and 28.4-fold, respectively, relative to D614G in BTI-HC, translating to massive antigenic distances of 5.7, 5.7, and 4.8 AU, respectively, with similar escape magnitudes in BTI-HIV (Figure 4B). These data highlight the relentless antigenic evolution of SARS-CoV-2 and the limited capacity of existing hybrid immunity to neutralize highly divergent contemporary strains.

## 4. Discussion

In this study, we comprehensively characterized SARS-CoV-2-specific antibody responses in vaccinated and unvaccinated individuals with or without HIV infection during the BA.4/5 and BF.7-dominated epidemic wave. Our findings demonstrate that in people living with HIV, standardized antiretroviral therapy is associated with robust hybrid immunity-induced functional antibody responses, including binding antibodies, neutralizing activity, and Fc-effector functions, which are quantitatively and qualitatively comparable to those in HIV-negative individuals. However, primary infection without prior vaccination correlates with profoundly limited antibody responses in this population, highlighting the critical importance of COVID-19 vaccination for protecting PLWH against circulating and emerging SARS-CoV-2 variants.

A key finding of our study is that PLWH receiving long-term suppressive ART achieved CD4^+^ T cell counts predominantly above 350 cells/μL, consistent with successful immune reconstitution (Figure 2A). Notably, despite significantly lower absolute CD4^+^ T cell counts compared to healthy controls, BTI-HIV participants generated RBD-binding IgG, neutralizing antibody, and ADCC responses that were indistinguishable from those in BTI-HC (Figure 2B and Figure 3). This observation aligns with previous reports suggesting that restored CD4^+^ T cell function, may be the primary determinant of vaccine responsiveness in well-controlled HIV infection [13,14]. Our data extend these findings to the context of breakthrough infection with highly immune-evasive Omicron subvariants, reinforcing that effective ART restores sufficient immunological competence to support robust hybrid immunity, conferred by COVID-19 vaccination followed by BF.7 or BA.4/5 breakthrough infection.

Our data also reveal a stark contrast between breakthrough and primary infection outcomes in PLWH. While BTI-HIV participants mounted robust functional antibody responses, PI-HIV individuals exhibited markedly diminished RBD-binding antibodies, neutralizing titers, and ADCC activity against all tested variants. Importantly, because CD4^+^ T cell counts were well-preserved and comparable across our PLWH groups, the severely blunted humoral responses in the PI-HIV cohort are primarily attributable to the absence of prior vaccine exposure rather than inherent HIV-induced immunodeficiency. This finding underscores that natural infection alone, even with contemporary Omicron variants, cannot substitute for vaccination in eliciting protective humoral immunity in ART-treated PLWH. PLWH who remain unvaccinated are likely to remain susceptible to reinfection and severe disease, particularly as SARS-CoV-2 continues to evolve antigenically. Our results strongly support prioritizing COVID-19 vaccination in HIV-positive populations, including those with well-controlled disease on ART, and may warrant more frequent booster recommendations given the rapid emergence of highly divergent variants.

An important observation in our study is the evidence of immunological imprinting in both BTI-HC and BTI-HIV cohorts. Despite breakthrough infection with BA.4/5 or BF.7, participants exhibited higher neutralizing titers against the ancestral D614G vaccine strain than against the infecting Omicron variant. This phenomenon, where prior antigenic exposure shapes the specificity of subsequent immune responses toward previously encountered epitopes, has been increasingly recognized in the context of sequential SARS-CoV-2 immunizations and infections [15,16]. Our extended variant panel analysis revealed progressive erosion of neutralization breadth across all cohorts, with the most pronounced escape observed for XDV, KP.2, and KP.3. While BTI-HC and BTI-HIV maintained marginal advantages over PI-HIV in neutralizing these contemporary variants, the absolute titers were substantially compromised. This finding aligns with recent reports demonstrating that KP.2 and KP.3 confer enhanced escape from vaccine and breakthrough infection-elicited neutralizing antibodies [17].

Beyond neutralization, we evaluated ADCC as a correlate of antibody-mediated protection. The preservation of robust Fc-effector functions in BTI-HIV, comparable to BTI-HC and significantly exceeding PI-HIV, has important implications for vaccine efficacy. Non-neutralizing antibody functions, including ADCC, antibody-dependent cellular phagocytosis (ADCP), and complement activation, have been associated with protection against SARS-CoV-2 infection and disease severity, particularly when neutralizing titers are suboptimal [18,19,20]. The maintenance of these effector mechanisms in vaccinated PLWH suggests that vaccines may provide partial protection against emerging variants even when neutralizing activity is compromised by antigenic drift.

Our study has several limitations. First, the limited sample size from a single-center hospital inherently introduces potential selection bias. Our carefully selected PLWH group (strictly on stable ART with suppressed viral loads) may not fully represent the broader HIV population. Additionally, while the sample size was adequate for detecting major group-wise differences, it restricts statistical power; therefore, non-significant findings between subgroups should be interpreted with caution, and complex multivariable adjustments for unmeasured residual confounders (e.g., baseline immune status or host genetics) could not be fully executed. Second, given the cross-sectional observational design, our findings only support associations rather than definitive causal inferences between clinical status and antibody generation. Furthermore, this design precludes assessment of antibody kinetics and durability; longitudinal follow-up would be valuable to determine the persistence of functional antibody responses and the impact of subsequent booster doses. Third, our cohorts were restricted to individuals with mild infection; whether similar patterns apply to those with severe COVID-19 or advanced HIV disease remains to be determined. Although comparing mild and severe cases would be informative, assembling matched severe cohorts is currently hindered by the rarity of severe COVID-19 in vaccinated, ART-suppressed PLWH and profound confounding from highly heterogeneous immune histories. Finally, our variant panel did not include all currently circulating strains; continued surveillance against emerging variants, including XFG, BA.3.2.2, LP.8.1, and XEC, is essential to determine whether patterns of immune imprinting and variant escape persist with continued antigenic evolution.

## 5. Conclusions

In summary, our findings demonstrate that suppressive ART enables PLWH to mount hybrid immunity via three doses of inactivated COVID-19 vaccination followed by BA.4/5 or BF.7 breakthrough infection, with functional antibody responses comparable to HIV-negative individuals. However, primary infection without vaccination elicits severely compromised humoral immunity. Immunological imprinting restricts the maturation of variant-specific responses in vaccinated individuals, and the most recent Omicron sublineages have evolved substantial escape from existing hybrid immunity. These results support the prioritization of updated COVID-19 vaccines containing contemporary variant antigens for HIV-positive populations and highlight the ongoing vulnerability of unvaccinated PLWH to antigenically divergent strains.

## Figures and Tables

**Table 1:** Clinical characteristics of the three cohorts used in this study.

	BTI-HC (*n* = 25)	BTI-HIV (*n* = 20)	PI-HIV (*n* = 10)
Male (%)	15 (60%)	13 (65%)	4 (40%)
Age [years, median (IQR)]	34 (30.5–45.5)	35 (26–46.5)	37.5 (34.3–37.3)
**CD4^+^ T cell counts [cells/mL, median (IQR)]**	**774 (629–939)**	**551 (474–636)**	**503 (475–630)**
Vaccine manufacturer **(*****n*****, %)** CoronaVac (Sinovac Life Sciences BBIBP-CorV (Beijing Institute of Biological Products)	21 (84%) 4 (16%)	16 (64%) 9 (36%)	-
Time interval between the 1st and 2nd dose [days, median (IQR)]	23 (21–32)	25 (22–29)	-
Time interval between the 2nd and 3rd dose [months, median (IQR)]	8 (6–9)	7 (6–9)	-
**Time interval between the 3rd dose and infection [months, median (IQR)]**	**13 (10–13)**	**10 (9–11)**	-
Time interval between infection and blood collection [days, median (IQR)]	29 (27–31)	29 (28–32)	30 (27–31)
ART duration [years, median (IQR)]	-	3.3 (2.5–4.1)	3.6 (2.2–5.9)
Current ART regimen			
NNRTI-based	-	9 (45%)	4 (40%)
INSTI-based	-	11 (55%)	6 (60%)

Bolded values indicate statistical significance. Abbreviations: BTI, breakthrough infection; HC, health controls; HIV, human immunodeficiency virus; PI, primary infection; IQR, interquartile range; ART, antiretroviral treatment; NNRTIs, non-nucleoside reverse transcriptase inhibitors: efavirenz (EFV)- or ainuovirine (ANV)-based regimens; INSTIs, integrase strand transfer inhibitors: dolutegravir (DTG)- or bictegravir (BIC)-based regimens. All regimens included one (lamivudine) or two NRTIs (tenofovir disoproxil fumarate/lamivudine or tenofovir alafenamide fumarate/emtricitabine) as backbone.

## Data Availability

Data was obtained from Beijing Ditan Hospital, Capital Medical University, and is available upon reasonable request from the corresponding author, Hongxin Zhao, by e-mail (drzhao66@ccmu.edu.cn).
